# The Land of Pluck

**Published:** 1891-08

**Authors:** Mary Mapes Dodge


### THE LAND OF PLUCK.

### A QUAINT AND QUEER BIT OF EARTH WHICH IS CALLED HOLLAND.

Far over the sea is a famous little country generally known as Holland ; but that name, even if it mean Hollow land, or How land ? does not describe it half so well as this—The Funny Land of Pluck.

Verily, a queerer bit of earth was never shone upon by the sun nor washed by the tide. It is the oddest, funniest country that ever raised its head from the waves (and, between ourselves, it does not quite do that), the most topsy turvy landscape, the most amphibious spot in the universe—as the man in the moon can't deny—the chosen butt of the elements, and good naturedly the laughing stock of mankind. Its people are the queerest and drollest of all the nations, and yet so plucky, so wise and resolute and strong that "beating the Dutch" has become a by-word for expressing the limits of mortal performance.

As for the country, for centuries it was not exactly anywhere—at least, it objected to staying long, just the same, in any one place. It may be said to have lain around loose on the waters of a certain portion of Europe, playing peek a boo with its inhabitants ; now coming to the surface here and there to attend to matters, then taking a dive for change of scene—and a most disastrous dive it often proved.

Rip Van Winkle himself changed less between his great sleeping and waking than Holland has altered many a time between sunset and dawn. All its permanence and resoluteness seems to have been soaked out of it, or rather to have filtered from the land into the people. Every field hesitates whether to turn into a pond or not, and the ponds are always trying to leave the country by the shortest cut. One would suppose that under this condition of things the only untroubled creatures would be turtles and ducks ; but no, strangest and most mysterious of all, every living thing in Holland appears to be thoroughly placid and content. The Dutch mind, so to speak, is at once anti-dry and waterproof.

Little children run about in fields where once their grandfathers sailed over the billows ; and youths and maidens row their pleasure boats where once their ancestors played "tag" among the haystacks. When the tide sweeps unceremoniously over Mynheer's garden he lights his pipe, takes his fishing rod and sits down on his back porch to try his luck. If his pet pond breaks loose and slips away he whistles, puts up a dam so that it cannot come back, and decides what crop shall be raised in its vacant place. None but the Dutch could live so tranquilly in Holland ; though, for that matter, if it had not been for the Dutch we may be sure there would have been by this time no Holland at all.

And yet this very Holland, besides holding its own place, has managed to gain a foothold in almost every quarter of the globe. An account of its colonies is a history in itself. In the East Indies alone it commands twenty-four millions of persons—*Mary Mapes Dodge in December St. Nicholas.*

